# JMJD1A, H3K9me1, H3K9me2 and ADM expression as prognostic markers in oral and oropharyngeal squamous cell carcinoma

**DOI:** 10.1371/journal.pone.0194884

**Published:** 2018-03-28

**Authors:** Lucas de Lima Maia, Gabriela Tonini Peterle, Marcelo dos Santos, Leonardo Oliveira Trivilin, Suzanny Oliveira Mendes, Mayara Mota de Oliveira, Joaquim Gasparini dos Santos, Elaine Stur, Lidiane Pignaton Agostini, Cinthia Vidal Monteiro da Silva Couto, Juliana Dalbó, Aricia Leone Evangelista Monteiro de Assis, Anderson Barros Archanjo, Ana Maria Da Cunha Mercante, Rossana Veronica Mendoza Lopez, Fábio Daumas Nunes, Marcos Brasilino de Carvalho, Eloiza Helena Tajara, Iúri Drumond Louro, Adriana Madeira Álvares-da-Silva

**Affiliations:** 1 Programa de Pós-Graduação em Biotecnologia, Universidade Federal do Espírito Santo, Vitória, Espírito Santo, Brazil; 2 Escola Multicampi de Ciências Médicas do Rio Grande do Norte, Universidade Federal do Rio Grande do Norte, Caicó, Rio Grande do Norte, Brazil; 3 Departamento de Anatomia Patológica, Hospital Heliópolis, São Paulo, São Paulo, Brazil; 4 Centro de Investigação Translacional em Oncologia, Instituto do Câncer do Estado de São Paulo, São Paulo, Brazil; 5 Departamento de Patologia Bucal, Faculdade de Odontologia, Universidade de São Paulo, São Paulo, São Paulo, Brazil; 6 Laboratório de Biologia Molecular, Hospital Heliópolis, São Paulo, São Paulo, Brazil; 7 Departamento de Biologia Molecular, Faculdade de Medicina, São José do Rio Preto, São Paulo, Brazil; Beijing Cancer Hospital, CHINA

## Abstract

**Aims:**

Jumonji Domain-Containing 1A (JMJD1A) protein promotes demethylation of histones, especially at lysin-9 of di-methylated histone H3 (H3K9me2) or mono-methylated (H3K9me1). Increased levels of H3 histone methylation at lysin-9 (H3K9) is related to tumor suppressor gene silencing. *JMJD1A* gene target Adrenomeduline (ADM) has shown to promote cell growth and tumorigenesis. JMJD1A and ADM expression, as well as H3K9 methylation level have been related with development risk and prognosis of several tumor types.

**Methods and results:**

We aimed to evaluate JMJD1A, ADM, H3K9me1 and H3K9me2expression in paraffin-embedded tissue microarrays from 84 oral and oropharyngeal squamous cell carcinoma samples through immunohistochemistry analysis. Our results showed that nuclear JMJD1A expression was related to lymph node metastasis risk. In addition, JMJD1A cytoplasmic expression was an independent risk marker for advanced tumor stages. H3K9me1 cytoplasmic expression was associated with reduced disease-specific death risk. Furthermore, high H3K9me2 nuclear expression was associated with worse specific-disease and disease-free survival. Finally, high ADM cytoplasmic expression was an independent marker of lymph node metastasis risk.

**Conclusion:**

JMJD1A, H3K9me1/2 and ADM expression may be predictor markers of progression and prognosis in oral and oropharynx cancer patients, as well as putative therapeutic targets.

## Introduction

Despite all advances in the understanding of molecular mechanisms involved in tumor development and progression, as well as new treatment protocols, head and neck squamous cell carcinoma (HNSCC) is still the sixth cause of death and cancer related morbidity, with over 600,000 new cases diagnosed every year [1,2,3].

HNSCC is a complex disease, caused by multiple factors such as smoking and drinking habits, HPV infection, dietary and genetic factors. It is also a diverse disease in relation to clinical presentation, treatment response and prognosis. In contrast with such diversity, there are common features that can lead to local and regional recurrence, as well as predict patient survival to the disease. The main prognostic factor for HNSCC is lymph node metastasis, decreasing by 50% patient survival chances. [4, 5]. Presence of tumor hypoxia is another important prognostic factor [6]

Tumor cell response to hypoxia involves activation of over 100 genes [7]. Currently, little is known about the epigenetic modulation that results from HIF system transcriptional activation [8]. However, such changes probably include epigenetic histone modifications [9].

The protein Jumonji Domain-Containing 1A (JMJD1A, JHDM2A or KDM3A) is regulated by HIF1a under hypoxic conditions. *JMJD1A* gene is activated via its hypoxia response element in the promoter region, resulting in demethylation of genes that help cell adaptation in low oxygen conditions [10]. Demethylation occurs at lysin and arginine residues of histones H3 in an oxygen-dependent reaction that needs Fe (II) ion and α-ketoglutarate as cofactors [11]. This can alter tumor cell behavior due to chromatin structural changes, gene expression and DNA repair [9,12].

Epigenetic regulation of gene expression through histone methylation has an important role in diverse biological processes, including cell cycle control, DNA damage response, cellular stress response, embryogenesis and cell differentiation [13, 14, 15]. Histone methylation changes are related to cancer due to its influence in tumor phenotype, such as differentiation, apoptosis and treatment response. Histone H3 methylation at residue 9 (H3K9) is specifically associated with transcriptional repression due to induction of heterochromatin formation and tumor suppressor gene silencing in various types of cancer [16, 17].

Adrenomedulin (ADM) is one of JMJD1A’s targets. *ADM* gene product, under normal conditions, is multifunctional, playing roles in cellular processes such as regulation of proliferation, differentiation, migration, growth, anti-apoptosis, angiogenesis, immunesuppression and hypoxia, suggesting its role in carcinogenesis [18, 19].

Histone methylation levels, as well as expression of JMJD1A and ADM have been associated with development and prognosis of several tumor types, such as colorectal [15, 20, 21], nasopharyngeal [22], hepatocellular [17, 23], renal [13, 24, 25]. Its role in HNC is still a matter of debate.

Therefore, we have aimed to study the association of JMJD1A, histones mono and di-methylated (H3K9me1 e H3K9me2, respectively) and ADM with clinicopathological features and prognosis of patients with HNC or cancer of the oral and oropharyngeal cavities.

## Material and methods

### Ethics

This study was approved by the Committee of Ethics in Research of the Heliópolis Hospital (CEP # 619) and a written informed consent was obtained from all patients enrolled.

### Samples

Samples were collected by the Head and Neck Genome Project (GENCAPO), a collaborative consortium created in 2002 with more than 50 researchers from 9 institutions in São Paulo State, Brazil, whose aim is to develop clinical, genetic and epidemiological analysis of HNSCC. In this study, 84 tumoral tissue samples were obtained and used for immunohistochemical analysis of the JMJD1A, within a total of 84 patients with oral and oropharyngeal squamous cell carcinomas, surgically treated at the Head and Neck Surgery Department of Heliópolis Hospital, São Paulo, Brazil, during the period of January/2002 to December/2008. The clinical follow-up was at least 24 months after surgery. Previous surgical or chemotherapic treatment, distant metastasis, no removal of cervical lymph nodes and positive surgical margins were exclusion criteria. Histopathological slides were reviewed by a senior pathologist to confirm the diagnosis and select appropriate areas for immunohistochemical analysis. Tumors were classified according to the TNM system (7^rd^ edition) [26].

Among analyzed individuals, mean age was 54.2 years (df ±10.3) being 72 (86%) for men and 12 (14%) for women. According to tumor anatomical sites, 64 (76%) were in the oral and 20 (24%) in the oropharyngeal cavity (Table 1; S1 Table).

**Table 1:** Epidemiological and prognostic features.

Features	Total	
	No.	(%)
**Gender**		
Female	12	14.3
Male	72	85.7
**Age, yr**		
mean 54.2, df±10,3		
**Smoker**	64	76.2
**Alcohol user**	48	57.1
**Tumor sites**		
Oral cavity	64	76.2
Oropharingeal	20	23.8
**Tumor size (T)** ^¥^		
T1, T2, T3	54	64.3
T4	30	35.7
**Lymph node (N)** ^¥^		
Negative	34	40.5
Positive	50	59.5
**Tumor stage**		
I, II, III	41	48.8
IV	43	51.2
**Disease relapse**		
No	27	32.1
Yes	55	65.5
Not available^§^	2	2.4
**Disease specific death**		
No	31	36.9
Yes	46	54.8
Not available^§^	7	8.3
**Total**	**84**	**100.0**

^¥^ TNM classification 7th edition.

^§^ Not available (not considered in the statistical calculations).

### Tissue microarray

Tissue microarrays were made as previously described [27] using buffered formalin-fixed paraffin-embedded tissue sections from 84 primary oral and oropharyngeal squamous cell carcinomas treated at the Head and Neck Surgery Department of Heliópolis Hospital, Saão Paulo, SP. Each slide was examined by a pathologist who marked the entire tumor circumference with a pen, after which, two 1mm diameter cylinders were punched from each block and reembedded in recipient paraffin blocks by a tissue microarrayer (Beecher Instruments^®^, Silver Spring, MD, USA). Sections were then taken from TMA, mounted in microscope slides, tissue microarray slides were evaluated by HE to confirm that tumor representative areas were extracted from all blocks. Every step of the process was supervised by 2 independent experienced pathologists, and after both of them approved the TMA procedure, IHC was performed on the slides.

### Immunohistochemistry

Anti-JMJD1A mouse monoclonal antibody (1:400)(107234, Abcam^®^), Anti- Histone H3 –Mono methyl K9 rabbit monoclonal antibody (1:600)(9045, Abcam^®^), Anti- Histone H3 –Di methyl K9 monoclonal mouse monoclonal antibody (1:800)(1220, Abcam^®^) and Anti-Adrenomedullin rabbit polyclonal antibody)(1:200) (69117, Abcam^®^) were used in the immunohistochemistry reaction using REVEAL Polimer-HRP, mouse/rabbit (Spring Bioscience), according to the manufacturer’s protocol [28–30]. Positive and negative controls (absence of primary antibody) were used for reaction quality control. Sample scoring was performed by semi-quantitative microscopic analysis, considering the number of stained tumor cells and signal intensity. Two spots were evaluated for each sample and a mean score was calculated. Considering the percentage of immune-positive tumor cells, a score of 1 was given when ≤10% of cells were positive; 2 when 11–50% of cells were positive and 3 when >50% of cells were positive. Signal intensity was scored as negative (0), weak (1), moderate (2) and strong (3). Both scores were multiplied [31, 32, 33] and the resulting score was used to categorize expression of the proteins as negative (0), positive low (1–3) and positive high (≥3). Two pathologists/investigators analyzed the slides, with no prior knowledge or discussion about the cases. Afterwards, the independent reports were compared for concordance, in which a 98.1% concordance rate was obtained. Non-concordant results were reanalyzed together in order to achieve a consensus between the 2 investigators.

### Statistical analysis

The chi square and Fisher exact tests were used for association analysis and confirmation was obtained by the Lilliefors test (significance considered when p<0.05). Multivariate logistic regression was used to obtain odds ratio (OR) and confidence intervals (CI ≥95%). Survival was calculated by the number of months between surgery and death for each patient or the last appointment in case the patient was alive. To calculate disease-free survival, the time endpoint was the date of disease relapse. The Kaplan-Meier model was used for survival analysis, using the Wilcoxon p-value and the Cox Proportional Hazards to adjust p-values and obtain hazard ratio (HR). Statistical calculations were performed using the IBM SPSS STATISTICS^®^ v. 20, 2011 softwares.

## Results

### Positive relationship between JMJD1A expression with lymph node status and tumor stage

**Fig 1:**
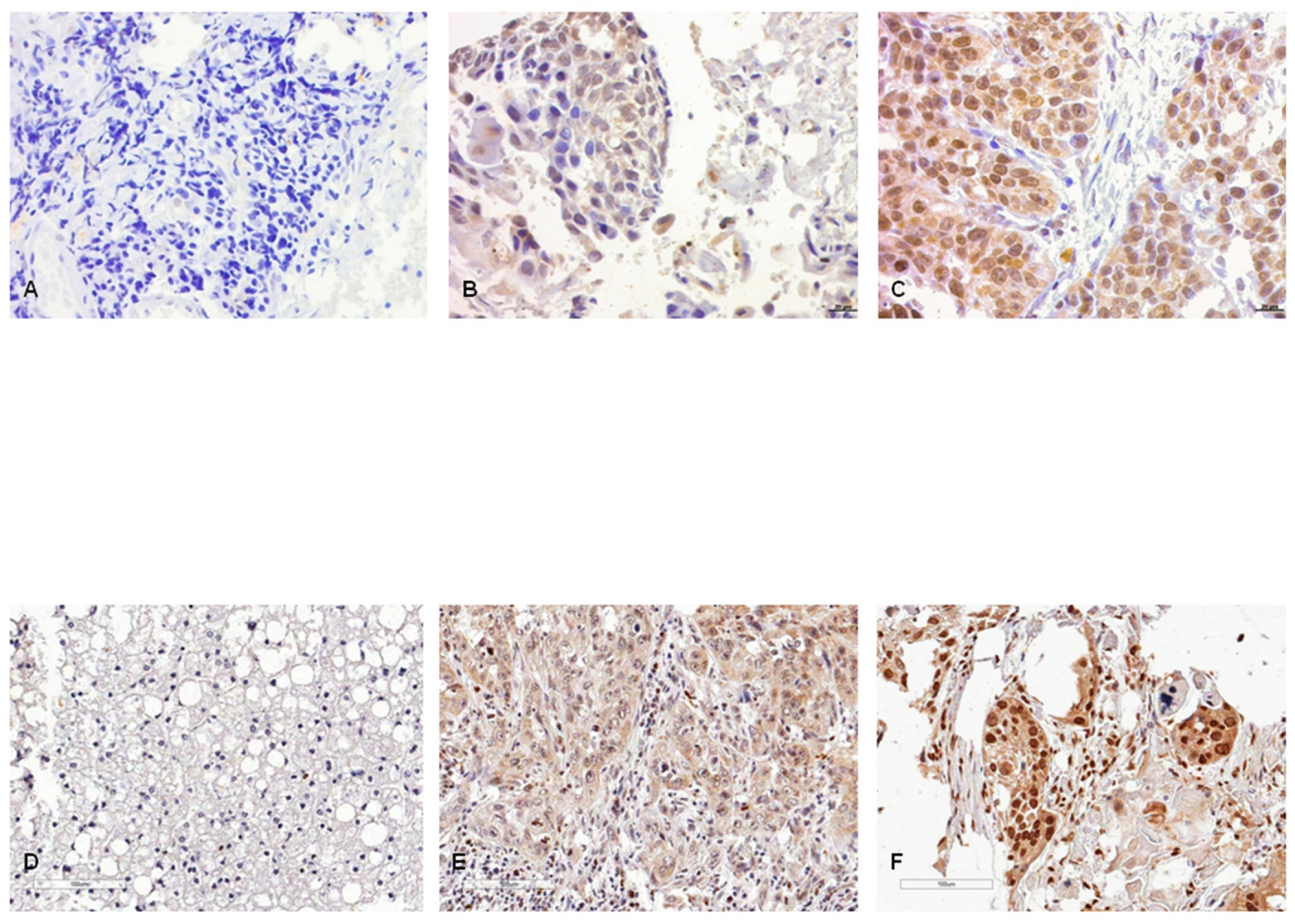
Immunohistochemistry. **(A)** Negative JMJD1A nuclear and cytoplasmic expression. **(B)** Low JMJD1A nuclear and cytoplasmic expression. **(C)** High JMJD1A nuclear and cytoplasmic expression. **(D)** Negative JMJD1A nuclear and cytoplasmic expression. **(E)** Low H3K9me1 nuclear and cytoplasmic expression. **(F)** High H3K9me1 nuclear and cytoplasmic expression. The scale bar indicates 10μμM. Magnification was 400x.

JMJD1A nuclear expression positivity was studied in 84 tumors, of which 27 were negative (32.1%), 21 were low positive (25.0%) and 29 were highly positive (34.6%). Regarding JMJD1A cytoplasmic expression, 11 were negative (13.1%), 59 (70.3%) were low positive and only 7 (8.3%) were high positive (Fig 1A, 1B and 1C; S2 Table). Seven of the 84 cases (8.3%) could not be analysed.

Positive JMJD1A nuclear expression showed a significant association with tumor stage and lymph-node status (p = 0.033 and p = 0.001, respectively, Table 2). Multivariate analysis showed that positive JMJD1A nuclear expression was an independent marker for lymph-node positivity, yielding an approximately 10-fold increased risk (OR = 10.086, CI = 2.02–50.35, Table 3). Moreover, JMJD1A nuclear expression levels did not show a significant relationship with tumor characteristics (Table 2).

**Table 2:** Clinical and pathological tumor features and their association with JMJD1A expression, according to cell localization.

Features	JMJD1A expression									
	Nuclear					Cytoplasmic				
	Negative		Positive		*p*	Negative		Positive		*p*
	No.	(%)	No.	(%)		No.	(%)	No.	(%)	
**Tumor size (T)** ^¥^										
T1, T2, T3	20	74.0	30	60.0	0.317	11	100.0	39	59.1	0.007
T4	7	26.0	20	40.0		0	0.0	27	40.9	
**Lymph node (N)** ^¥^										
Negative	18	66.7	13	26.0	<0.001	10	90.9	21	31.8	<0.001
Positive	9	33.3	37	74.0		1	9.1	45	68.2	
**Tumor stage**										
I, II, III	18	66.7	20	40.0	0.033	10	90.9	28	42.4	0.003
IV	9	33.3	30	60.0		1	9.1	38	57.6	
**Disease relapse**										
No	7	25.9	14	28.0	0.764	3	45.5	18	27.3	0.954
Yes	20	74.1	34	68.0		8	54.5	46	69.7	
Not available^§^	0	0.0	2	4.0		0	0.0	2	3.0	
**Disease specific death**										
No	9	33.3	18	36.0	0.946	3	27.3	24	36.4	0.729
Yes	14	51.9	29	58.0		6	54.5	37	56.0	
Not available^§^	4	14.8	3	6.0		2	18.2	5	7.6	
**Total**	**27**	**35.0**	**50**	**65.0**		**11**	**14.3**	**66**	**85.7**	

^¥^ TNM classification 7th edition.

^§^ Not available (not considered in the statistical calculations).

**Table 3:** Multivariate analysis of the relationship between lymph node status and JMJD1A and ADM expression.

Features	Multivariate Analysis							
	Lymph nodes (N) ^¥^		Tumor stage ^¥^		Disease specific death		Disease relapse	
	OR (IC 95%)	*p*	OR (IC 95%)	*p*	OR (IC 95%)	*p*	OR (IC 95%)	*p*
**JMJD1A Nuclear expression**								
Negative	1							
Positive	10.08 (2.02–50.35)	0.005						
**JMJD1A Cytoplasmic expression**								
Negative	1		1					
Positive	10.74 (0.63–182.30)	0.100	0.09 (0.00–0.93)	0.043				
**H3K9me1 Cytoplasmic expression**								
Low			1		1			
High			0.25 (0.03–1.70)	0.157	0.06 (0.00–0.79)	0.032		
**H3K9me2 nuclear expression**								
Low					1		1	
High					3.50 (0.82–14.81)	0.089	4.15 (0.99–17.31)	0.050
**ADM Cytoplasmic expression**								
Low	1							
High	9.16 (1.14–73.47)	0.037						
**Tumor size (T)** ^¥^								
T1, T2, T3	1				1		1	
T4	8.82 (1.60–48.41)	0.012			3.88 (0.99–15.14)	0.050	3.78 (0.92–15.50)	0.064
**Necrosis**								
Absent	1		1					
Present	4.81 (0.73–31.76)	0.102	4.81 (0.73–31.76)	0.102				
**Smoking**								
No	1							
Yes	13.46 (2.14–84.52)	0.006						
**Alcoholism**								
No					1		1	
Yes					3.92 (1.09–14.09)	0.036	2.53 (0.79–8.08)	0.117
**Age**								
≤ 55					1			
> 55					2.89 (0.84–9.87)	0.090		
**Anatomical site**								
Oral cavity	1							
Oropharynx	8.46 (1.36–52.49)	0.022						

^¥^ TNM classification 7th edition.

Positive JMJD1A cytoplasmic expression was significantly associated with tumor size (p = 0.007), lymph-node status (p<0.001) and tumor stage (p = 0.003, Table 2). Multivariate analysis showed that positive JMJD1A cytoplasmic expression was an independent marker for tumor stage, yielding an reduced risk (OR = 0.092, CI = 0.009–0.930, Table 3). In addition, JMJD1A cytoplasmic expression levels did not show a significant relation with tumor characteristics (Table 2).

### H3K9 monomethylation expression in cytoplasm reduces disease specific death risk

H3K9me1 nuclear expression positivity was studied in 84 tumors, of which 16 (19.1%) were negative, 44 (52.4%) were low positive and 19 (22.6%) were highly positive. Regarding H3K9me1 cytoplasmic expression, 12 (14.3%) were negative, 58 (69.1%) were low positive and only 9 (10.7%) were highly positive (Fig 1D, 1E and 1F; S2 Table). Five of the 84 cases (5.9%) could not be analysed due to IHC failure.

Positive H3K9me1 nuclear expression did not show a significant association with tumor characteristics (Table 4).

**Table 4:** Clinical and pathological tumor features and their association with H3K9me1 expression, according to cell localization.

Features	H3K9me1 expression									
	Nuclear					Cytoplasmic				
	Negative		Positive		*p*	Negative		Positive		*p*
	No.	(%)	No.	(%)		No.	(%)	No.	(%)	
**Tumor size (T)** ^¥^										
T1, T2, T3	11	68.8	39	61.9	0.774	7	63.6	42	62.7	0.952
T4	5	31.2	24	38.1		4	36.4	25	37.3	
**Lymph node (N)** ^¥^										
Negative	6	37.5	25	39.7	0.873	4	36.4	26	38.8	0.877
Positive	10	62.5	38	60.3		7	63.6	41	61.2	
**Tumor stage**										
I, II, III	8	50.0	29	46.0	0.787	5	54.5	31	46.3	0.960
IV	8	50.0	34	54.0		6	36.4	36	53.7	
**Disease relapse**										
No	3	18.8	18	28.6	0.365	0	0.0	21	31.3	0.028
Yes	13	81.2	38	60.3		10	90.9	40	59.7	
Not available^§^	0	0.0	7	11.1		1	9.1	6	9.0	
**Disease specific death**										
No	5	31.3	22	34.9	0.766	1	9.1	26	38.8	0.039
Yes	10	62.5	30	47.6		9	81.8	30	44.8	
Not available^§^	1	6.2	11	17.5		1	9.1	11	16.4	
**Total**	**16**	**20.3**	**63**	**79.7**		**11**	**14.1**	**67**	**85.9**	

^¥^ TNM classification 7th edition.

^§^ Not available (not considered in the statistical calculations).

Positive H3K9me1 cytoplasmic expression showed a significant association with disease relapse (p = 0.028) and disease specific death (p = 0.039, Table 4). Multivariate analysis showed that positive H3K9me1 cytoplasmic expression was an independent marker for disease specific death (OR = 0.068; IC = 0.006–0.793, Table 3). In addition, cytoplasmic expression levels did not show a significant relation with tumor characteristics (Table 4).

### H3K9 dimethylathion influences disease free and disease specific survival

**Fig 2:**
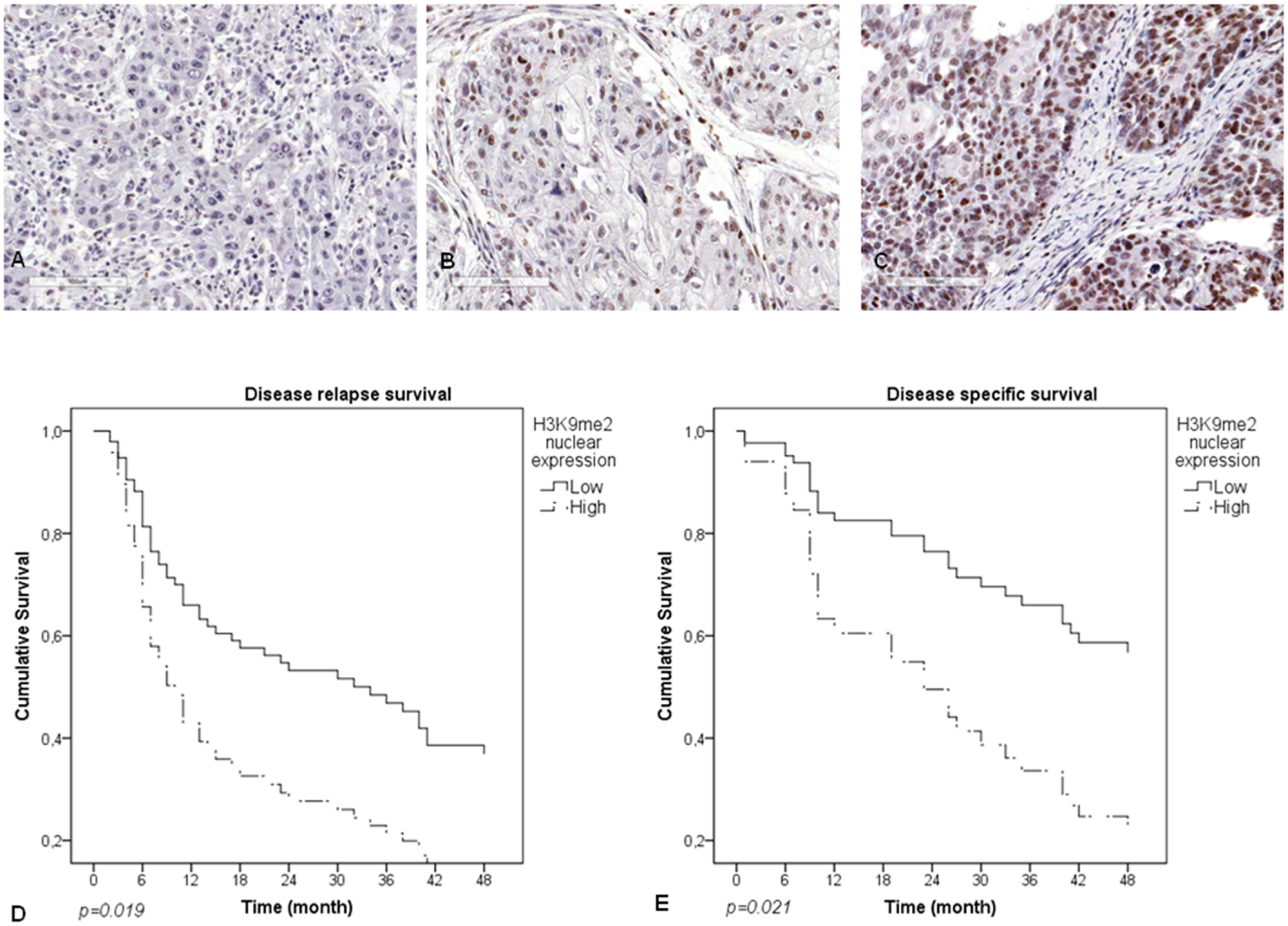
Survival plots and immunohistochemistry. **(A)** Negative H3K9me2 nuclear expression **(B)** Low H3K9me2 nuclear expression. **(C)** High H3K9me2 nuclear expression. (**D**) Disease relapse survival according to H3K9me2 nuclear expression. **(E)** Disease specific survival according to H3K9me2 nuclear expression. The scale bar indicates 10μμM. Magnification was 400x.

H3K9me2 nuclear expression positivity was studied in 84 tumors, of which 7 (8.3%) were negative, 50 (59.5%) were low positive and 25 (29.8%) were highly positive. However, H3K9me2 cytoplasm expression was positive only in 3 (3.5%) cases (Fig 2A, 2B and 2C; S2 Table). Two of the 84 cases (2.4%) could not be analysed.

Positive H3K9me2 nuclear expression did not show a significant association with tumor characteristics (Table 5). Although multivariate analysis did not show a signifcant relationship between cytoplasmic H3k9me2 expression and systemic disease relapse, the data suggests a tendency of association between them (p = 0.050, Table 3).

**Table 5:** Clinical and pathological tumor features and their association with H3K9me2 and ADM expression, according to cell localization.

Features	H3K9me2 expression					ADM expression				
	Nuclear					Cytoplasmic				
	Negative		Positive		*p*	Negative		Positive		*p*
	No.	(%)	No.	(%)		No.	(%)	No.	(%)	
**Tumor size (T)** ^¥^										
T1, T2, T3	5	71.4	47	62.7	0.645	2	66.7	49	62.8	0.892
T4	2	28.6	28	37.3		1	33.3	29	37.2	
**Lymph node (N)** ^¥^										
Negative	3	42.9	29	38.7	0.828	1	33.3	31	39.7	0.824
Positive	4	57.1	46	61.3		2	66.7	47	60.3	
**Tumor stage**										
I, II, III	5	71.4	34	45.3	0.249	2	66.7	36	46.2	0.598
IV	2	28.6	41	54.7		1	33.3	42	53.8	
**Disease relapse**										
No	0	0.0	21	28.0	0.177	2	66.7	19	24.4	0.197
Yes	6	85.7	48	64.0		1	33.3	51	65.4	
Not available^§^	1	14.3	6	8.0		0	0.0	8	10.2	
**Disease specific death**										
No	1	14.3	26	34.7	0.394	1	66.7	25	32.1	0.558
Yes	5	71.4	38	50.7		2	33.7	40	51.3	
Not available^§^	1	14.3	11	14.6		0	0.0	13	16.6	
**Total**	**7**	**8.5**	**75**	**91.5**		**3**	**3.7**	**78**	**96.3**	

^¥^ TNM classification 7th edition.

^§^ Not available (not considered in the statistical calculations).

In contrast, H3K9me2 protein expression showed an association with both disease-free survival (p = 0.019, Table 6, Fig 2D) and disease-specific survival (p = 0.021, Table 6, Fig 2E). Multivariate analysis showed that high nuclear H3K9me2 protein expression decreases disease free survival by more than 2-fold (HR = 2.034; CI = 1.12–3.67, Table 6), whereas high nuclear expression decreased disease specific survival by approximately 3-fold (HR = 2,620; CI = 1,156–5,938, Table 6). We have attempted to compare our survival data with the one present in TCGA survival database, however no success was achieved because of either lack of information in the TCGA database or no significant statistical results after the comparisons.

**Table 6:** Multivariate analysis of the relationship between disease relapse and disease specific survival and H3k9me2 expression and tumor size.

Variáveis	Cox Proportional		Cox Proportional	
	Disease relapse survival		Disease specific survival	
	HR (IC 95%)	*p*	HR (IC 95%)	*p*
**H3K9me2 Nuclear expression**				
Low	1		1	
High	2.034(1.126–3.676)	0.019	2.620(1.156–5.938)	0.021
**Tumor size (T)** ^¥^				
T1, T2,T3	1		1	
T4	1.993(1.113–3.570)	0.020	2.444 (1.128–5.296)	0.024

^¥^ TNM classification 7th edition.

### ADM expression as an independent marker of lymph node positivity

**Fig 3:**
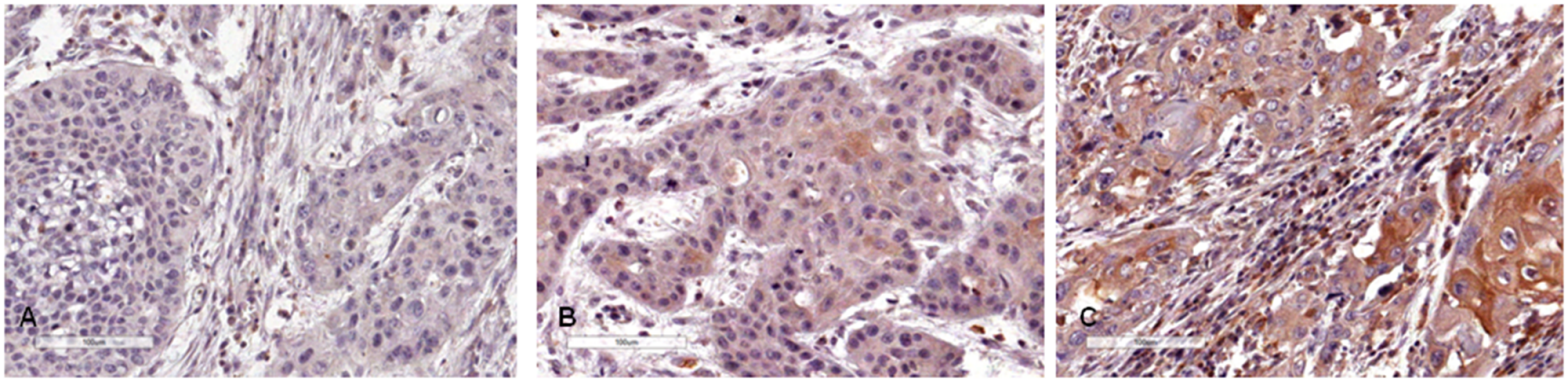
Immunohistochemistry. **(A)** Negative ADM cytoplasmic expression **(B)** Low ADM cytoplasmic expression. **(D)** High ADM cytoplasmic expression. The scale bar indicates 10μμM. Magnification was 400x.

ADM did not show nuclear expression. In contrast, ADM cytoplasmic expression was negative in 3 cases (3.6%), low positive in 64 (76.2%) and highly positive in 14 (16.6%) (Fig 3A, 3B and 3C; S2 Table). Three of the 84 cases could not be analysed (3.6%).

Positive ADM cytoplasmic expression did not show a significant association with tumor characteristics (Table 5). ADM cytoplasmatic expression levels showed a significant relationship with lymph-node status (p = 0.037, Table 5). Multivariate analysis showed that strong ADM cytoplasmic expression was an independent marker for lymph-node positivity, yielding an approximately 9-fold increased risk (OR = 9.167; CI = 1.14–73.47, p = 0.037, Table 3).

## Discussion and conclusions

JMJD1A protein promotes demethylation of histones, especialy at lysin-9 of di-methylated histone H3 (H3K9me2) or mono-methylated (H3K9me1) [11]. Histone demethylation alters chromatin structure resulting in gene expression changes, DNA repair, replication [12], as well as cell differentiation [13]. The ADM protein has its expression altered by the action of JMJD1A [23]. The JMJD1A and ADM expression, as well as the histone H3K9 methylation level, have been related to development and prognosis of diverse tumor types [13–25].

**Fig 4:**
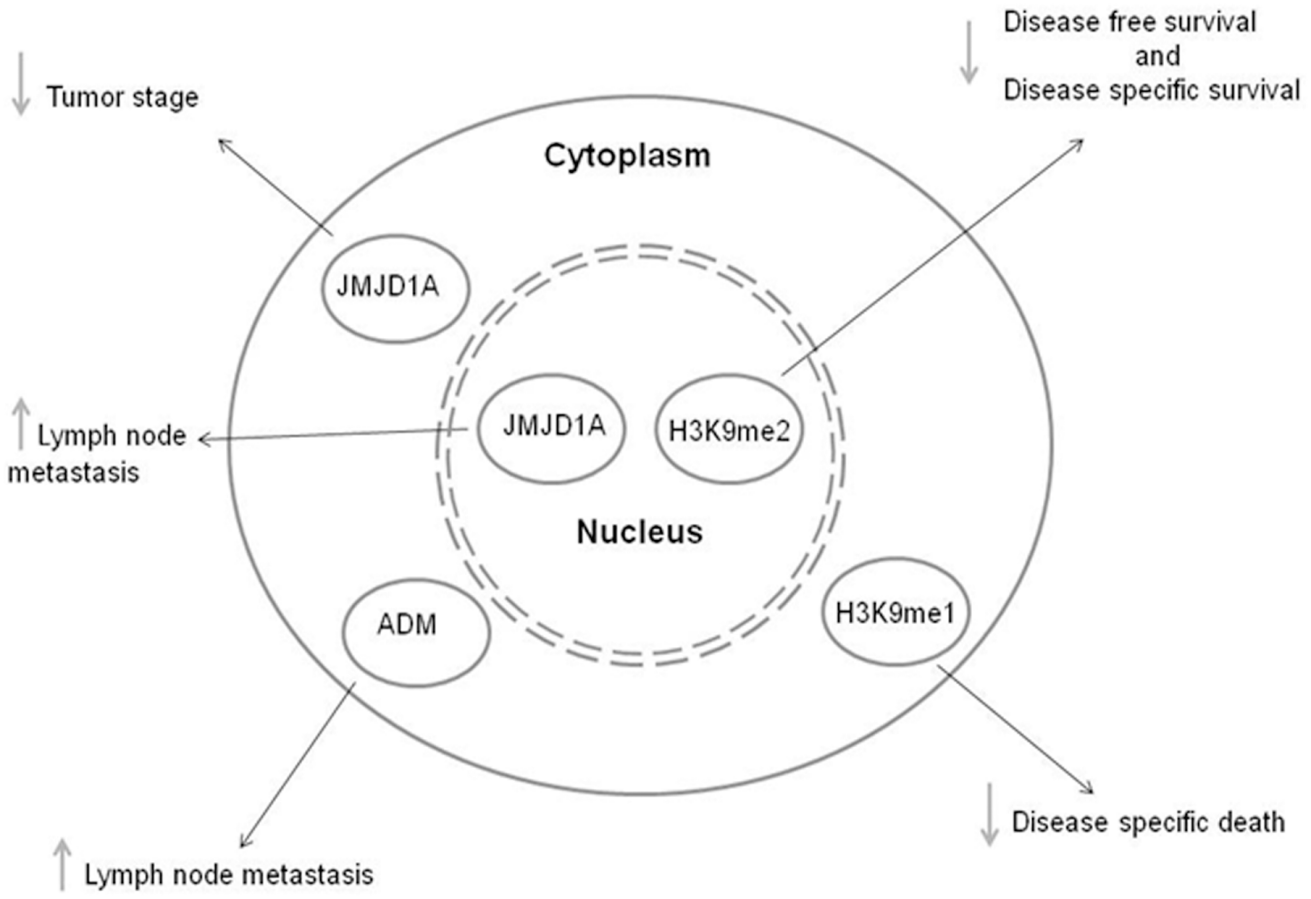
Influence of JMJD1A, H3K9me1, H3K9me2 and ADM in clinicopathological tumor features and patient survival in HNC. Nuclear JMJD1A expression increases lymph node positivity risk. In contrast, cytoplasmic expression decreases advanced tumor stage risk. Positive cytoplasmic H3K9me1 protein expression reduces disease specific death. High nuclear H3K9me2 expression causes a worse diseas-specific and disease-free survival. High cytoplasmic ADM expression is related with high lymph node metastasis risk.

Our results showed that positive JMJD1A nuclear expression is a worse prognostic factor because increases lymph node metastasis risk by over 10-fold (Fig 4). Colorectal cancer studies have correlated high JMJD1A expression with an augmented risk for lymph node positivity by over 6-fold [20]. Other studies with cervical, gastric and nasopharyngeal cancer have reported an association JMJD1A expression and an increased risk of lymph node metastasis [22, 34, 35].

Our results suggest that JMJD1A cytoplasmic expression is related with less aggressive tumor stages. JMJD1A protein levels in certain cytoplasmic locations is dependent upon cell growth rate and Hsp90 chaperone activity [36, 37, 38], which also interferes with JMJD1A stability and activity [36]. Kasiolius et al. (2014) showed that JMJD1A deficiency in rats resulted in cytoskeleton abnormalities, which in turn was associated with metastatic potential, more aggressive tumors and decreased global survival and disease-free survival in breast cancer patients [39]. This study also showed an association between ADM protein expression and lymph node metastasis, so that high expression increases lymph node metastasis risk by 9 fold.

ADM was also associated with increased lymph node metastasis risk in ovary cancer [40]. Moreover, ADM high expression was associated with lymphatic angiogenesis and lymph node metastasis risk [19]. In addition, high ADM expression is associated with cell proliferation, tumor cell survival and tumor cell escape from immune surveillance [18]. In breast cancer, ADM expression was associated with distant metastasis and worse prognosis [41]. Higher ADM mRNA expression level was observed in patients with positive lymph nodes, suggesting its role in lymph node metastasis [42], therefore being a predictor of such in breast cancer [43].

Our results related with H3K9m1 and H3K9m2 showed that H3K9me1 cytoplasmic expression is associated with a lower risk of disease specific death, whereas nuclear H3K9me2 expression is related with a worse disease-free and disease-specific survival. Pre-methylation of H3 histone into monomethylated H3 (H3k9me1) is a cytoplasmic process mediated by cytoplasmic Prdm3 and Prdm16 histone methyl transferases. Methylated histones are likely to act upon chromatin compactation and gene expression silencing. In such regions, H3K9me1 will be converted into H3K9me2 and H3K9me3 by the SUV39h enzyme [44]. Therefore, high cytoplasmic expression of H3K9me1 suggests a lower histone methylation in the tumor and a lower risk of disease specific death.

High nuclear H3K9me2 protein expression is related with a lower survival, such that after 30 months of follow up, 60% of high nuclear protein expression had decreased due to the disease, as compared to 30% of the ones with low expression. A study about salivary adenoid cystic carcinoma revealed that patients with highly methylated histones showed a lower survival when compared to patients with weak methylation [14]. A gastric cancer study observed that high levels of methylated H3K9 were associated with a lower survival, suggesting it as a potential independent marker of worse survival in gastric cancer [45].

In a similar fashion, the present study has observed that the intensity of nuclear H3K9me2 expression was significantly associated with disease-free survival. In 30 months, over 60% of patients with high protein expression had shown tumor relapse, whereas 20% of patients with low expression had relapsed. Additionally, high expression of methylated H3K9 was associated with worse survival in acute myeloid leukemia [46]. Changes in Histone H3 expression levels may predict relapse and survival in lung cancer patients [47].

According to our results and with abundant literature data, we believe that histone methylation levels results in a worse prognosis due to silencing of tumor suppressor genes. Therefore, our results support a role of histone methylation patterns in HNSCC, as well as of JMJD1A and ADM, suggesting them as candidate biomarkers of prognosis in this cancer.

## Supporting information

S1 Table Clinical and pathological tumor features per patient. (DOCX)

S2 Table Expression of JMJD1A, H3K9me1, H3K9me2 and ADM per patient. (DOCX)
