# Supplementary material for: JMJD1A, H3K9me1, H3K9me2 and ADM expression as prognostic markers in oral and oropharyngeal squamous cell carcinoma
Source: PLoS One. 2018 Mar 28;13(3):e0194884. doi: 10.1371/journal.pone.0194884 (PMC5874045; doi:10.1371/journal.pone.0194884)
Supplement: S1 Table — (DOCX) [file pone.0194884.s001.docx]

| S1 Table: | Clinical and pathological tumor features per patient. | | | | | | | | | |
| --- | --- | --- | --- | --- | --- | --- | --- | --- | --- | --- |
| Sample (patients) | **Gender** | **Age**  **(yr)** | **Smoker** | **Alcohol user** | **Tumor sites** | **Tumor stage** | **Tumor size (T)** | **Lymph node (N)** | **Disease specific death** | **Disease relapse** |
| 1 | Female | >55 | Yes | No | oral cavity | III | pT2 | Positive | No | No |
| 2 | Male | ≤ 55 | Yes | Yes | oral cavity | II | pT2 | Negative | No | Yes |
| 3 | Male | ≤ 55 | Yes | Yes | oral cavity | III | pT3 | Negative | Yes | Yes |
| 4 | Female | ≤ 55 | Yes | Yes | oropharynx | IV | pT4 | Positive | Yes | Yes |
| 5 | Male | >55 | No | Yes | oral cavity | IV | pT3 | Positive | Yes | Yes |
| 6 | Male | ≤ 55 | Yes | No | oral cavity | III | pT3 | Negative | No | Yes |
| 7 | Male | ≤ 55 | Yes | No | oropharynx | III | pT3 | Positive | No | No |
| 8 | Male | >55 | Yes | Yes | oral cavity | II | pT2 | Negative | No | No |
| 9 | Male | ≤ 55 | Yes | No | oropharynx | IV | pT4 | Positive | Yes | Yes |
| 10 | Male | ≤ 55 | Yes | Yes | oral cavity | IV | pT4 | Positive | Yes | Yes |
| 11 | Male | >55 | Yes | No | oral cavity | II | pT2 | Negative | Yes | Yes |
| 12 | Male | ≤ 55 | Yes | No | oropharynx | IV | pT2 | Positive | Yes | Yes |
| 13 | Male | >55 | No | No | oropharynx | II | pT2 | Negative | Yes | Yes |
| 14 | Male | ≤ 55 | Yes | Yes | oral cavity | IV | pT4 | Positive | Yes | Yes |
| 15 | Male | ≤ 55 | Yes | Yes | oral cavity | IV | pT4 | Negative | Yes | Yes |
| 16 | Male | ≤ 55 | Yes | Yes | oropharynx | IV | pT4 | Positive | ------ | No |
| 17 | Female | >55 | No | No | oral cavity | IV | pT4 | Negative | ------ | No |
| 18 | Male | >55 | No | Yes | oral cavity | IV | pT4 | Positive | Yes | Yes |
| 19 | Male | ≤ 55 | Yes | Yes | oral cavity | II | pT2 | Negative | Yes | Yes |
| 20 | Male | ≤ 55 | Yes | Yes | oral cavity | III | pT3 | Positive | Yes | Yes |
| 21 | Male | >55 | Yes | Yes | oropharynx | III | pT2 | Positive | Yes | Yes |
| 22 | Male | >55 | Yes | Yes | oral cavity | III | pT3 | Negative | Yes | Yes |
| 23 | Male | ≤ 55 | No | Yes | oral cavity | IV | pT4 | Positive | ------ | Yes |
| 24 | Male | >55 | No | No | oral cavity | IV | pT4 | Negative | No | Yes |
| 25 | Female | >55 | Yes | No | oral cavity | II | pT2 | Negative | Yes | Yes |
| 26 | Male | ≤ 55 | Yes | Yes | oral cavity | IV | pT2 | Positive | Yes | Yes |
| 27 | Female | ≤ 55 | No | No | oral cavity | III | pT2 | Positive | Yes | Yes |
| 28 | Male | ≤ 55 | Yes | No | oropharynx | III | pT3 | Negative | Yes | Yes |
| 29 | Male | >55 | Yes | Yes | oral cavity | IV | pT2 | Positive | Yes | Yes |
| 30 | Male | ≤ 55 | Yes | Yes | oral cavity | IV | pT4 | Negative | Yes | Yes |
| 31 | Male | ≤ 55 | Yes | No | oral cavity | II | pT2 | Negative | No | No |
| 32 | Male | ≤ 55 | Yes | Yes | oral cavity | III | pT2 | Positive | No | No |
| 33 | Male | ≤ 55 | Yes | Yes | oral cavity | IV | pT4 | Positive | No | No |
| 34 | Female | ≤ 55 | Yes | Yes | oropharynx | IV | pT4 | Positive | Yes | Yes |
| 35 | Male | >55 | No | No | oral cavity | II | pT2 | Negative | No | Yes |
| 36 | Male | >55 | No | Yes | oropharynx | III | pT2 | Positive | No | Yes |
| 37 | Male | >55 | Yes | No | oral cavity | IV | pT4 | Positive | ------- | Yes |
| 38 | Male | ≤ 55 | Yes | Yes | oral cavity | IV | pT4 | Positive | No | No |
| 39 | Female | >55 | Yes | No | oropharynx | IV | pT2 | Positive | Yes | Yes |
| 40 | Female | >55 | No | No | oral cavity | IV | pT4 | Positive | Yes | Yes |
| 41 | Female | >55 | No | No | oral cavity | II | pT2 | Negative | ------- | No |
| 42 | Male | ≤ 55 | Yes | Yes | oral cavity | IV | pT2 | Positive | No | No |
| 43 | Male | ≤ 55 | Yes | Yes | oral cavity | IV | pT4 | Positive | Yes | Yes |
| 44 | Male | ≤ 55 | Yes | No | oropharynx | IV | pT3 | Positive | Yes | Yes |
| 45 | Male | >55 | Yes | Yes | oral cavity | IV | pT4 | Positive | No | No |
| 46 | Male | ≤ 55 | No | No | oral cavity | IV | pT4 | Negative | No | No |
| 47 | Male | >55 | No | Yes | oral cavity | II | pT2 | Negative | Yes | Yes |
| 48 | Male | ≤ 55 | Yes | Yes | oral cavity | IV | pT4 | Positive | ------- | No |
| 49 | Male | ≤ 55 | Yes | Yes | oral cavity | III | pT1 | Positive | No | No |
| 50 | Male | >55 | Yes | No | oral cavity | IV | pT4 | Positive | Yes |  |
| 51 | Male | >55 | Yes | Yes | oral cavity | IV | pT2 | Positive | Yes | Yes |
| 52 | Male | >55 | No | No | oral cavity | II | pT2 | Negative | No | Yes |
| 53 | Male | ≤ 55 | Yes | Yes | oropharynx | I | pT1 | Negative | No | No |
| 54 | Female | >55 | No | No | oral cavity | IV | pT4 | Positive | No | Yes |
| 55 | Male | >55 | Yes | No | oral cavity | I | pT1 | Negative | No | No |
| 56 | Male | ≤ 55 | Yes | No | oral cavity | III | pT3 | Negative | Yes | Yes |
| 57 | Male | ≤ 55 | Yes | Yes | oral cavity | IV | pT4 | Positive | Yes | Yes |
| 58 | Male | >55 | Yes | Yes | oral cavity | IV | pT4 | Positive | Yes | Yes |
| 59 | Male | ≤ 55 | Yes | Yes | oral cavity | III | pT3 | Negative | No | Yes |
| 60 | Male | ≤ 55 | Yes | Yes | oropharynx | III | pT3 | Positive | Yes | Yes |
| 61 | Male | >55 | Yes | Yes | oropharynx | II | pT2 | Negative | Yes | Yes |
| 62 | Male | >55 | Yes | Yes | oral cavity | IV | pT4 | Positive | Yes | Yes |
| 63 | Male | ≤ 55 | Yes | Yes | oral cavity | IV | pT4 | Positive | No | No |
| 64 | Male | >55 | Yes | Yes | oral cavity | IV | pT4 | Positive | Yes |  |
| 65 | Female | ≤ 55 | Yes | No | oral cavity | IV | pT2 | Positive | Yes | Yes |
| 66 | Male | ≤ 55 | Yes | No | oropharynx | IV | pT3 | Positive | Yes | Yes |
| 67 | Male | >55 | Yes | No | oral cavity | IV | pT3 | Positive | No | No |
| 68 | Male | >55 | No | Yes | oral cavity | II | pT2 | Negative | Yes | Yes |
| 69 | Male | ≤ 55 | Yes | No | oral cavity | II | pT2 | Negative | No | No |
| 70 | Male | ≤ 55 | Yes | Yes | oral cavity | IV | pT4 | Positive | No | No |
| 71 | Male | >55 | Yes | Yes | oral cavity | II | pT2 | Negative | Yes | Yes |
| 72 | Male | >55 | No | No | oral cavity | III | pT3 | Negative | ------- | No |
| 73 | Male | >55 | No | No | oropharynx | IV | pT4 | Positive | Yes | Yes |
| 74 | Male | >55 | Yes | No | oral cavity | II | pT2 | Negative | No | Yes |
| 75 | Male | >55 | No | No | oropharynx | IV | pT3 | Positive | No | No |
| 76 | Male | ≤ 55 | Yes | No | oropharynx | III | pT3 | Negative | No | No |
| 77 | Male | >55 | Yes | Yes | oropharynx | III | pT3 | Positive | No | No |
| 78 | Male | >55 | Yes | Yes | oral cavity | IV | pT4 | Positive | Yes | Yes |
| 79 | Male | >55 | Yes | Yes | oral cavity | IV | pT3 | Positive | Yes | Yes |
| 80 | Male | ≤ 55 | No | No | oral cavity | II | pT2 | Negative | No | No |
| 81 | Male | ≤ 55 | Yes | Yes | oral cavity | III | pT3 | Positive | Yes | Yes |
| 82 | Female | ≤ 55 | Yes | No | oral cavity | I | pT1 | Negative | No | No |
| 83 | Male | >55 | Yes | Yes | oral cavity | III | pT2 | Positive | Yes | Yes |
| 84 | Male | ≤ 55 | Yes | Yes | oral cavity | III | pT3 | Negative | Yes | Yes |

------- Not available (not considered in the statistical calculations).
