# Supplementary material for: JMJD1A, H3K9me1, H3K9me2 and ADM expression as prognostic markers in oral and oropharyngeal squamous cell carcinoma
Source: PLoS One. 2018 Mar 28;13(3):e0194884. doi: 10.1371/journal.pone.0194884 (PMC5874045; doi:10.1371/journal.pone.0194884)
Supplement: S2 Table — (DOCX) [file pone.0194884.s002.docx]

| S2 Table: | | Expression of JMJD1A, H3K9me1, H3k9me2 and ADM per patient. | | | | | | | |
| --- | --- | --- | --- | --- | --- | --- | --- | --- | --- |
| Sample (patients) | | **Nuclear JMJD1A expression** | **Cytoplasmic JMJD1A expression** | **Nuclear H3K9me1 expression** | **Cytoplasmic H3K9me1 expression** | **Nuclear H3K9me2 expression** | **Cytoplasmic H3K9me2 expression** | **Nuclear ADM expression** | **Cytoplasmic ADM expression** |
| 1 | Negative | Positive low | Negative | Positive low | Positive low | Negative | Negative | Positive high |  |
| 2 | Negative | Positive low | Negative | Positive low | Negative | Negative | Negative | Positive low |  |
| 3 | Negative | Negative | Negative | Negative | Negative | Negative | --------- | --------- |  |
| 4 | Negative | Positive low | --------- | --------- | Negative | Negative | Negative | Negative |  |
| 5 | Positive low | Positive high | Negative | Positive high | --------- | Negative | Negative | Positive high |  |
| 6 | Negative | Positive low | Positive high | Positive low | Positive high | Negative | Negative | Positive low |  |
| 7 | Negative | Positive low | Positive low | Positive low | Positive low | Positive low | Negative | Positive low |  |
| 8 | Positive low | Positive low | Positive low | Positive low | Positive low | Negative | Negative | Positive low |  |
| 9 | --------- | --------- | Positive low | Negative | Positive low | Negative | Negative | Positive low |  |
| 10 | Positive low | Positive low | Positive low | Positive low | Positive low | Negative | Negative | Positive low |  |
| 11 | Negative | Positive low | Negative | Positive low | Negative | Positive low | Negative | Positive low |  |
| 12 | Negative | Positive low | Positive high | Negative | Positive low | Negative | Negative | Positive low |  |
| 13 | Negative | Negative | Positive low | Negative | Positive low | Negative | Negative | Positive low |  |
| 14 | Positive low | Positive low | Positive low | Positive low | Positive low | Negative | Negative | Positive low |  |
| 15 | Negative | Positive low | Positive low | Positive low | Positive low | Negative | Negative | Positive low |  |
| 16 | Negative | Positive low | Negative | Positive low | Positive low | Negative | Negative | Positive high |  |
| 17 | Negative | Positive low | Positive low | Positive low | Positive high | Negative | Negative | Positive low |  |
| 18 | Positive low | Positive low | Negative | Positive high | Positive low | Negative | Negative | Positive high |  |
| 19 | Positive high | Positive low | Positive high | Positive low | Positive high | Negative | Negative | Positive low |  |
| 20 | Positive high | Positive low | Positive high | Positive low | Positive high | Negative | Negative | Positive low |  |
| 21 | Negative | Positive low | Negative | Positive low | Negative | Negative | Negative | Positive low |  |
| 22 | --------- | --------- | --------- | --------- | --------- | --------- | --------- | --------- |  |
| 23 | Positive high | Positive low | Positive low | Positive low | Positive high | Negative | Negative | Positive high |  |
| 24 | Positive high | Positive low | Positive low | Positive low | Positive low | Negative | Negative | Positive low |  |
| 25 | --------- | --------- | --------- | --------- | --------- | --------- | Negative | Positive low |  |
| 26 | Negative | Positive low | Negative | Positive high | Positive low | Negative | Negative | Positive high |  |
| 27 | Positive low | Positive low | --------- | ---------. | Negative | Positive low | --------- | --------- |  |
| 28 | Negative | Positive low | Negative | Positive low | Positive low | Negative | Negative | Positive low |  |
| 29 | --------- | --------- | Positive low | Positive low | Positive high | Negative | Negative | Positive low |  |
| 30 | Negative | Positive low | Positive low | Positive low | Positive low | Negative | Negative | Positive low |  |
| 31 | Negative | Positive high | Negative | Positive low | Positive low | Negative | Negative | Positive low |  |
| 32 | Positive high | Positive low | Positive low | Positive low | Positive low | Negative | Negative | Positive high |  |
| 33 | Positive high | Positive low | Positive high | Positive low | Positive low | Negative | Negative | Positive low |  |
| 34 | Positive high | Positive high | Negative | Positive high | Positive low | Negative | Negative | Positive low |  |
| 35 | Positive high | Positive low | Positive high | Positive low | Positive high | Negative | Negative | Positive low |  |
| 36 | Positive low | Positive low | Positive low | Negative | Positive low | Negative | Negative | Positive low |  |
| 37 | Positive high | Positive low | Positive high | Positive low | Positive high | Negative | Negative | Positive high |  |
| 38 | --------- | --------- | Positive low | Positive high | Positive high | Negative | Negative | Positive high |  |
| 39 | Positive high | Positive high | Positive low | Positive low | Positive high | Negative | Negative | Positive low |  |
| 40 | Positive low | Positive low | Positive high | Negative | Positive high | Negative | Negative | Positive low |  |
| 41 | Negative | Negative | Positive low | Positive low | Positive low | Negative | Negative | Positive low |  |
| 42 | Positive high | Positive low | Positive low | Positive low | Positive low | Negative | Negative | Positive low |  |
| 43 | Negative | Positive low | Positive low | Positive high | Positive high | Negative | Negative | Positive low |  |
| 44 | Positive high | Positive low | Positive high | Positive low | Positive high | Negative | Negative | Positive low |  |
| 45 | --------- | --------- | Positive high | Positive low | Negative | Negative | Negative | Positive low |  |
| 46 | Negative | Positive low | Positive high | Positive low | Positive low | Negative | Negative | Positive high |  |
| 47 | Positive low | Positive low | Positive low | Positive low | Positive low | Negative | Negative | Positive low |  |
| 48 | Positive high | Positive low | Positive low | Positive low | Positive low | Negative | Negative | Positive low |  |
| 49 | Positive low | Positive low | Positive high | Positive low | Positive low | Negative | Negative | Positive low |  |
| 50 | Positive high | Positive low | Positive high | Positive low | Positive low | Negative | Negative | Positive low |  |
| 51 | Positive low | Positive low | Positive low | Positive low | Positive low | Negative | Negative | Positive low |  |
| 52 | Positive high | Positive low | Positive low | Positive high | Positive low | Negative | Negative | Positive high |  |
| 53 | Positive high | Positive low | Positive low | Positive low | Positive high | Negative | Negative | Positive low |  |
| 54 | Positive low | Positive low | Negative | Positive low | Positive low | Negative | Negative | Positive low |  |
| 55 | Positive high | Positive low | Positive low | Positive low | Positive low | Negative | Negative | Positive low |  |
| 56 | Positive low | Negative | Positive low | Positive low | Positive low | Negative | Negative | Positive low |  |
| 57 | Positive low | Positive low | Positive low | Negative | Positive low | Negative | Negative | Positive low |  |
| 58 | Positive high | Positive low | Positive low | Negative | Positive high | Negative | Negative | Positive low |  |
| 59 | --------- | --------- | Positive low | Positive low | Positive high | Negative | Negative | Positive low |  |
| 60 | Positive high | Positive high | Positive low | Positive low | Positive low | Negative | Negative | Positive low |  |
| 61 | Positive high | Positive low | Positive low | Positive low | Positive low | Negative | Negative | Positive low |  |
| 62 | Positive high | Positive low | Positive high | Positive low | Positive high | Negative | Negative | Positive low |  |
| 63 | Positive high | Positive low | Positive low | Positive low | Positive low | Negative | Negative | Positive low |  |
| 64 | Positive high | Positive low | Positive high | Positive low | Positive high | Negative | Negative | Positive low |  |
| 65 | Positive low | Positive low | Positive low | Negative | Positive low | Negative | Negative | Positive low |  |
| 66 | Positive low | Positive low | Positive low | Positive low | Positive high | Negative | Negative | Positive low |  |
| 67 | Positive high | Positive low | Negative | Positive high | Positive low | Negative | Negative | Positive low |  |
| 68 | Negative | Negative | Negative | Negative | Positive low | Negative | Negative | Positive low |  |
| 69 | Negative | Negative | Positive low | Positive low | Positive low | Negative | Negative | Negative |  |
| 70 | Positive low | Positive low | Positive low | Positive low | Positive low | Negative | Negative | Positive low |  |
| 71 | Negative | Negative | Positive low | Positive low | Positive low | Negative | Negative | Positive low |  |
| 72 | Negative | Negative | Positive low | Positive high | Positive low | Negative | Negative | Positive low |  |
| 73 | Positive low | Positive low | Positive low | Positive low | Positive high | Negative | Negative | Positive low |  |
| 74 | Negative | Positive high | Positive low | Negative | Positive high | Negative | Negative | Positive low |  |
| 75 | Positive low | Positive low | Positive high | Positive low | Positive high | Negative | Negative | Positive low |  |
| 76 | Positive low | Negative | --------- | --------- | Positive low | Negative | Negative | Positive low |  |
| 77 | Negative | Positive low | Positive low | Positive low | Positive low | Negative | Negative | Negative |  |
| 78 | Positive high | Positive low | Negative | Positive low | Positive low | Negative | Negative | Positive high |  |
| 79 | Positive high | Negative | Positive high | Positive low | Positive low | Negative | Negative | Positive low |  |
| 80 | Positive high | Positive low | Positive high | Positive low | Positive low | Negative | Negative | Positive high |  |
| 81 | Positive high | Positive low | Positive low | Positive low | Positive low | Negative | Negative | Positive high |  |
| 82 | Negative | Negative | Positive high | Positive low | Positive low | Negative | Negative | Positive low |  |
| 83 | Positive high | Positive high | Positive low | Positive low | Positive high | Negative | Negative | Positive low |  |
| 84 | Positive low | Positive low | Positive low | Negative | Positive high | Negative | Negative | Positive low |  |

--------- Not available (not considered in the statistical calculations).
